# Small bowel obstruction secondary to left paraduodenal hernia: A case report and literature review

**DOI:** 10.1016/j.ijscr.2018.10.018

**Published:** 2018-10-12

**Authors:** Youssef Sleiman, Alaa El-Kheir, Melody El-Khoury, Omar Hamdach, Layla Ismail, Mustafa Allouch

**Affiliations:** a Department of Surgical Oncology, Institut Jules Bordet, Université Libre de Bruxelles (ULB), Lebanon; b Department of General and Digestive Surgery, Nini Hospital, Tripoli, Lebanon; c Department of Radiology, Nini Hospital, Tripoli, Lebanon

**Keywords:** CIH, congenital internal hernia, LPDH, left paraduodenal hernia, SBO, small bowel obstruction, CT, computed tomography, IMV, inferior mesenteric vein, Internal hernia, Left paraduodenal hernia, Landzert fossa, Intestinal obstruction

## Abstract

**Introduction:**

Internal hernias are uncommon and are defined by the protrusion of a viscus through intraperitoneal defects. Left paraduodenal hernias (LPDH) are the most common type of congenital internal hernia (CIH).

We report a clinical case of a patient with a small bowel obstruction (SBO) due to a LPDH.

**Case presentation:**

An elderly patient, with negative surgical history, presented to the emergency room with abdominal pain and vomiting. Clinical and radiologic investigations were consistent with SBO due to an internal hernia. During laparotomy, a large LPDH containing dilated small bowel loops was identified. The hernia contents were reduced and the opening was closed with non-absorbable sutures. The post-operative course was uneventful without signs of recurrence at follow up.

**Discussion:**

LPDH result from absence of retroperitoneal fixation of the descending colon mesentery. Usually, patients are asymptomatic and LPDH are incidental findings detected on imaging and/or during laparotomy. However, LPDH may lead to acute SBO therefore a high index of clinical suspicion and preoperative imaging is crucial to make an early diagnosis.

**Conclusion:**

CIH is a rare condition due to embryologic developmental abnormalities. Their diagnosis is based on pathognomonic findings on computed tomography scanner (CTscan). Surgical treatment is the mainstay treatment. Delay diagnosis of strangulated CIH is associated with significant morbidity andmortality.

## Highlights

• Congenital internal hernias are an infrequent condition resulting from the protrusion of abdominal organ through an intra-peritoneal defect.
• Left paraduodenal hernia is the most common types of congenital internal hernias.
• Landzert fossa is bounded by the fourth part of the duodenum from the right, the posterior peritoneum posteriorly, the inferior mesenteric vein, and left branches of the middle colic artery anteriorly.
• Laparotomy is mandated in cases of intestinal perforations, necrosis and hemodynamic instability.

## 1 Introduction

Congenital internal hernias (CIH) is an infrequent condition resulting from the protrusion of an abdominal organ through an intra-peritoneal foraminae. It can be natural and anatomical such as the Winslow foramen or abnormal resulting from embryological abnormalities such as gut malrotation or absence of retroperitoneal attachments (paraduodenal fossa, ileocecal fossa) [1,2].

CIH are responsible for 0.6–5.8% of the etiologies of small bowel obstruction (SBO) [1]. Left paraduodenal hernias (LPDH) represents the most common types of CIH and accounts for more than 40% of all cases [2]. The symptoms of LPDH are often non specific and can range from intermittent abdominal pain when the hernia is spontaneously reducing to acute surgical abdomen when there is strangulation and necrosis [3,4]. The diagnosis of LPDH is difficult as symptoms are often nonspecific and many clinicians are unfamiliar with this rare condition. High index of suspicion and good knowledge of the anatomy are requisite for clinical and radiological diagnosis of LPDH [3,4]. LPDH carry a reported lifetime risk of obstruction and bowel strangulation of around 50% with a mortality of 20% and higher [4].

We report a clinical case of an elderly patient with SBO due to a large LPDH and a brief literature review about the management of SBO secondary to LPDH. This work has been reported in line with the SCARE criteria [5].

## 2 Case presentation

**Fig. 1:**
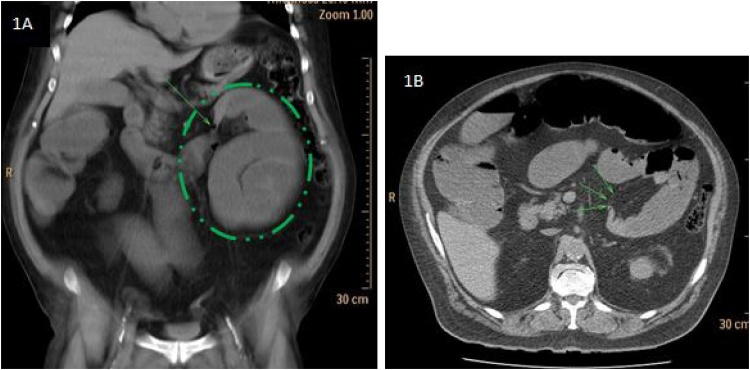
A and 1B: CT scan shows dilated small bowel loops with transition zone located left to the Treitz ligament (green arrow) and saclike appearance (green circle).

A 76-year-old male patient presented to our emergency department with a 24 h history of gradual onset left upper quadrant abdominal pain associated with nausea, vomiting, anorexia, and obstipation. Patient had no medical history of abdominal surgery. On presentation, he was afebrile, dehydrated with normal heart rate and blood pressure. On clinical examination his abdomen was distended and tender upon palpation of left upper quadrant without guarding or rebound tenderness. White blood cell count and CRP were 10,500/mm³ (3500–11,000) and 10 mg/L (<10) respectively. He had a slight renal insufficiency with a creatinine level of 2.6 mg/dl (0.72–1.17).The rest of the blood tests (Electrolytes, hepatic function) were unremarkable. Abdominal X-rays study showed a cluster of small bowel loops in left upper quadrant with multiple air-fluid levels. Abdominal computed tomography scanner (CT scan) confirmed the diagnosis of SBO with transition zone left to the Treitz ligament (Fig. 1A and B) and showed a sac-like appearance in left paraduodenal fossa located posterior to the mesentery of the left colon and the left portion of the mesentery of the transverse colon measuring 30 cm (Fig. 1A) suggesting of a left paraduodenal hernia.

**Fig. 2:**
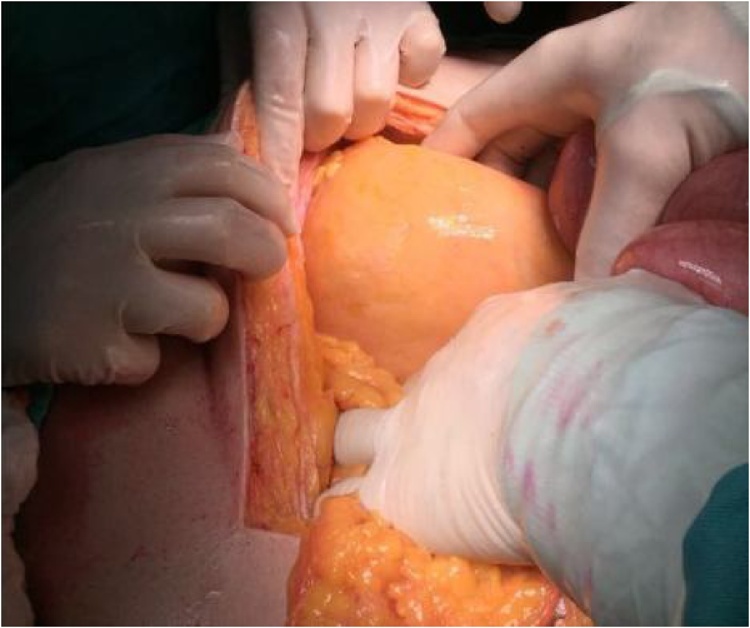
Intra-operative photograph showing the hernia sac in landzert fossa posterior to the descending mesocolon that contains dilated small bowel loops.

**Fig. 3:**
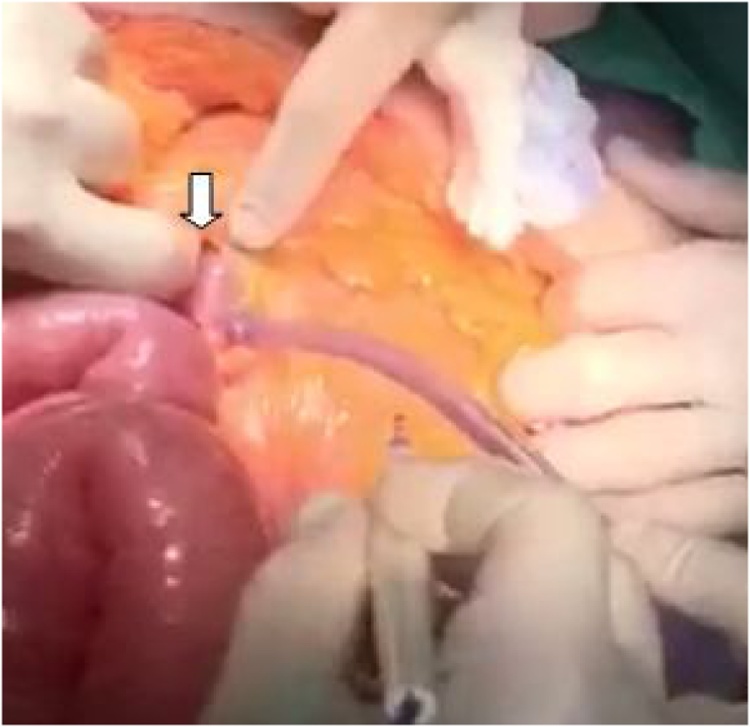
Intra-operative photograph showing the hernia orifice (white arrow).

Due to the high risk of strangulation, and the presence of symptoms, the patient was consented for exploratory laparotomy. At exploration, a huge hernia sac (Fig. 2) arising from a peritoneal opening (Fig. 3) located left to the Treitz ligament was identified. The sac contained dilated loops of small bowels.The hernia was reduced after widening of the orifice inferiorly and the defect was closed using non-resorbable sutures. The patient started oral feeding at day 2 post-operative after removing of the nasogastric tube and he was discharged home on 5th post-operative day. At 8 months follow-up, he is doing well without any complaints.

## 3 Discussion

LPDH are three times more common than the right paraduodenal hernias with an autopsy incidence of 2% [6,7]. LPHD are defined by the herniation of the small bowel postero-inferiorly into the left paraduodenal or Landzert fossa. Landzert fossa is bound by the fourth part of the duodenum from the right, the posterior parietal attachment of the descending colon mesentery posteriorly, the IMV, and the left branches of the middle colic artery anteriorly [8,9]. This peritoneal opening results from an embryological development abnormality related to the absence of fusion of both peritoneal folds, the visceral peritoneum of the posterior side of the mesentery of the descending colon and the left posterior parietal fold, during the 6th–11th week of gestation [6].

Male are affected 2–3 times more than females [7]. Although congenital, patients are usually diagnosed in the 4th–5th decades of life [6]. The delay in presentation and diagnosis is related to the degree of small bowel entrapment [3]. When the hernia is not incarcerated, patients may be asymptomatic or having an intermittent abdominal pain, but when there is strangulation, the patient presents with severe continuous pain associated with vomiting and obstipation. Clinical abdominal examination is positive for tenderness in the left upper quadrant and for rebound tenderness and guarding in case of bowel necrosis [3]. Approximately 50% of patients that are operated on in emergency from strangulated hernia remember to have presented previous nonspecific abdominal pain [3,10].

In most of reported cases, the diagnosis of LPDH is made during laparotomy [10]. However, pre-operative diagnosis by conventional imaging (x-Ray and CT scan) can be achieved in 43% of patients [10]. On abdominal x-Ray, LPDH appears as a clustering of small bowel loops in the left upper quadrant but imaging appearance is not specific. Abdominal CT scan is the imaging technique of choice for pre-operative diagnosis of all types of internal hernias. CT scan can depict pathognomonic findings by detecting an intestinal closed loop, by identifying the hernia defect, and by analyzing abnormal displacement of surrounding structures and key-vessels around the hernia orifice and the hernia sac. LPDH appears as cluster of intestinal loops with a saclike appearance in the left anterior para-renal space posterior to the mesentery of the transverse and descending colon with possible mass effect on the transverse colon or posterior stomach [2,10].The IMV and ascending left colic artery are landmarks situated at the anteromedial edge of Landzert fossa [2,10].

In our patient the suspicion of LPDH on CT scan led to the decision of urgent surgery as in case of acute presentations related to hernia strangulation, the reported mortality varies from 20 to 50% [10].A laparotomy was performed because of the abdominal distention. Laparotomy is also mandatory in all cases and obviously those of intestinal perforations, necrosis and hemodynamic instability [11]. Operative management is based on reduction of herniated small bowel loops, resection of ischemic intestinal segments and closure of the hernia orifice with non-absorbable sutures or mesh [3,4]. Excision of the hernia sac is not required [12]. Widening of the hernia orifice to reduce the hernia by performing an incision into an avascular area inferiorly to avoid injury of regional vessels may be necessary [3,4,7]. Division of the IMV on its base is advocated in very tight orifice [3,4,7].

## 4 Conclusion

LPDH has to be considered among the differential diagnosis of small bowel obstruction in a patient without abdominal surgical history. In such a case, urgent surgery is warranted due to the high risk of mortality in cases of hernia strangulation. Detailed knowledge of the anatomy, etiology and vascular landmarks allow the surgeons to accurately manage this type of hernia.

## Conflicts of interest

We have no conflict of interest to declare.

## Funding sources

The authors have no funding source to disclose for this work.

## Ethical approval

The submitted article is a case report, ethical approval has been exempted by our institution.

## Consent

Written informed consent was obtained from the patient for publication of this case report and accompanying images.

## Author contribution

Youssef Sleiman, Alaa El-Kheir and Omar Hamdach: paper concept, design, data collection and interpretation. Youssef Sleiman and Alaa El-Kheir: writing the manuscript. Layla Ismail and Melody El-Khoury: reviewing and editing. Mustafa Allouch was the operating surgeon and responsible for drafting and revising the article content and for the final approval of the manuscript prior to submission.

## Registration of research studies

Not applicable.

## Guarantor

Dr Mustafa Allouch

Nini Hospital, El-Maarad Street, Tripoli, Lebanon.

allouchmustafa@gmail.com.

Tel: 009613684906.

## Provenance and peer review

Not commissioned, externally peer reviewed.
